# Proteomic Profile of Carbonylated Proteins Screen Regulation of Apoptosis via CaMK Signaling in Response to Regular Aerobic Exercise

**DOI:** 10.1155/2018/2828143

**Published:** 2018-12-18

**Authors:** Wenfeng Liu, Li Li, Heyu Kuang, Yan Xia, Zhiyuan Wang, Shaopeng Liu, Dazhong Yin

**Affiliations:** ^1^Hunan Provincial Key Laboratory of Physical Fitness and Sports Rehabilitation, Hunan Normal University, Changsha, Hunan 410012, China; ^2^Department of Experimental and Clinical Pharmacology, University of Minnesota, Minneapolis, MN 55455, USA; ^3^School of Health & Kinesiology, Georgia Southern University, Statesboro, GA 30460, USA; ^4^Qingyuan People's Hospital, The Sixth Affiliated Hospital, Guangzhou Medical University, Guangzhou, Guangdong, China

## Abstract

To research carbonylated proteins and screen molecular targets in the rat striatum on regular aerobic exercise, male Sprague-Dawley rats (13 months old, n = 24) were randomly divided into middle-aged sedentary control (M-SED) and aerobic exercise (M-EX) groups (n = 12 each). Maximum oxygen consumption (VO_2max_) gradually increased from 50%–55% to 65%–70% for a total of 10 weeks. A total of 36 carbonylated proteins with modified oxidative sites were identified by Electrospray Ionization-Quadrupole-Time of Flight-Mass Spectrometer (ESI-Q-TOF-MS), including 17 carbonylated proteins unique to the M-SED group, calcium/calmodulin-dependent protein kinase type II subunit beta (CaMKII*β*), and heterogeneous nuclear ribonucleoprotein A2/B1 (Hnrnpa2b1), among others, and 19 specific to the M-EX group, ubiquitin carboxyl-terminal hydrolase isozyme L1 (UCH-L1), and malic enzyme, among others. Regular aerobic exercise improved behavioral and stereological indicators, promoted normal apoptosis (P < 0.01), alleviated carbonylation of the CaMKII*β* and Hnrnpa2b1, but induced carbonylation of the UCH-L1, and significantly upregulated the expression levels of CaMKII*β*, CaMKII*α*, and Vdac1 (*p* < 0.01) and Hnrnpa2b1 and UCH-L1 (*p* < 0.01), as well as the phosphoinositide 3-kinase/protein kinase B/mammalian target of rapamycin pathways (PI3K/Akt/mTOR) pathway-related genes Akt and mTOR. Regular aerobic exercise for 10 weeks (incremental for the first 6 weeks followed by constant loading for 4 weeks) enhanced carbonylation of CaMKII*β*, Hnrnpa2b1, and modulated apoptosis via activation of CaMK and phosphoinositide 3-kinase/protein kinase B/mTOR signaling. It also promoted normal apoptosis in the rat striatum, which may have protective effects in neurons.

## 1. Introduction

Posttranslational modifications including phosphorylation, glycosylation, and carbonylation alter the relative molecular mass and isoelectric point of proteins and, consequently, their activity and functions. Carbonyl stress refers to the production of reactive carbonyl species that exceeds the body's scavenging capacity, resulting in the carbonylation of proteins and other macromolecules and pathophysiological changes including accelerated aging [1] through formation of amyloid and neurofibrillary tangles, nuclear protein enrichment, and neuronal accumulation of lipofuscin pigment granules.

Toxic carbonyl compounds are generated in the body as byproducts of the reactions that form the major biological macromolecules—i.e., lipids, carbohydrates, and proteins. These compounds include unsaturated aldehydes and ketones related to oxidative stress such as 4-hydroxy-nonennal (HNE), acrolein, formaldehyde, and malondialdehyde as well as advanced glycation end products and free radicals produced by the degradation of sugars. Carbonylation leads to the formation of oxidized proteins; carbonyl-ammonia reactions cause intramolecular or intermolecular cross-linking, which affects normal protein structure and function [2–5]. Carbonylated proteins are considered as a general indicator of oxidative stress, in which reactive oxygen species accumulate in cells and cause irreversible damage to DNA, RNA, and lipids [6]. Oxidative stress has been implicated in a variety of neurological diseases including Alzheimer's disease, PD, and amyotrophic lateral sclerosis.

Lesions in different parts of the striatum can produce changes in muscle tone and involuntary movements such as dyskinesia in Parkinson's disease (PD) and chorea in Huntington's disease. Striatal dopamine content, as measured by positron emission tomography, is correlated with learning, hearing, and memory [7, 8]. On the other hand, learning and memory are thought to be dependent on the number of dopamine receptors in the striatum; continuous depletion of dopamine in the striatal system leads to dysfunction of memory or recognition in PD patients [9]. Impairment of striatal function such as through afferent modulation of dopaminergic neurons of the midbrain is an important marker of PD [10, 11].

Exercise is used in rehabilitation medicine to activate restorative biological processes. Long-term regular aerobic exercise has been shown to improve the body's antioxidant capacity, reduce damage caused by free radicals, suppress lipid peroxidation, and reduce lipofuscin deposition, thereby contributing to the maintenance of brain health and delaying brain aging. It was previously reported that long-term moderate-intensity exercise during middle age reduces the risk of PD development in later life [12–14].

The present study investigated whether regular aerobic exercise can prevent oxidative stress-associated protein carbonylation in middle-aged rats by peptide mass and fingerprinting analysis.

## 2. Materials and Methods

### 2.1. Experimental Animals and Ethics Statement

Specific pathogen-free 13-month-old male Sprague-Dawley (SD) rats (n=24, 693.21±68.85g) were supplied by the Animal Center of East Biotechnology Services Company (Changsha, Hunan, China; License number: Xiang scxk 2009-0012). Four rats were housed in each standard cage (3 cages/group) with free access to food and water. All animals were kept in an air-conditioned room maintained at a constant temperature of 20°C to 25°C with a relative humidity of 45% to 55%. Rats were subjected to a cycle of 12 h light and 12 h darkness. All animals were acclimated to laboratory conditions for 2 weeks, prior to the start of the experiment. A total of 24 rats were randomly assigned to two groups—middle-aged sedentary control group (M-SED, n=12) and aerobic exercise runner group (M-EX, n=12) by elderly weight. All animal procedures were approved by the local ethics committee (the Institutional Review Board of Hunan Normal University) and the Guidelines for Care and Use of Laboratory Animals (Washington (DC) 2011). Disposal of animals was done in accordance with “*The guidance on the care of laboratory animals*” (the provisions were issued in 2006 by the Ministry of Science and Technology of the People's Republic of China).

### 2.2. Exercise Protocol

The aerobic exercise regime used in this study was proposed by Koltai and Zhang [15], with reference to the exercise load standards of Bedford [16]. All animals in the M-SED and M-EX groups were first subjected to a 5-day adaptation period on a rat treadmill (slope gradient 0%, ZH-PT-1 Treadmill, Li Tai Bio-Equipment Co., Ltd., Hangzhou, Zhejiang, China). Adapted training was carried out at a speed of 10 m/min and a gradient of 0%, for a gradually increasing duration of time—10 min on the first day, 20 min on the second day, and 25 min on the third day. During this period, they were placed on a belt facing away from the electrified grid (0.6 mA intensity), twice a day. For the actual experiment, the M-EX group underwent daily training by running at 15 m/min (equivalent to approx. 50% to 60% peak oxygen uptake) [16], on a slope of 0°, for a duration of 15 min. During the first week at the start of the training, the exercise duration was increased gradually from 15 min to the second week 20 min, the third week 25 min, the fourth week 30 min, and the fifth and sixth week 35 min. The M-EX group underwent daily training by running at 20 m/min and maintained speed for a week (equivalent to approx. 65% to 70% peak oxygen uptake) (Bedford et al., 1979), on a slope of 0°, for a duration of 35 min during the seventh to tenth week. The acceleration of the treadmill was set such that, at about 3 min after the start of the training, the final speed of 20 m/min was achieved. To ensure that the animals completed the exercise regime, we used sound stimulation and a small wooden stick to stimulate the animals' tails, when necessary. We also used electrical stimulation to keep the rats at one-third distance on the treadmill runway. Animals were required to perform the training 6 days a week for a total of 10 weeks [17].

### 2.3. Tissue Specimen Collection and Analysis

On the day following the last exercise schedule, the rats were anesthetized with chloral hydrate (400 mg/kg, i.p.) and decapitated. The striatum (Stereotaxic Atlas of the Rat Brain; George Paxinos., 2005) [18] was excised from all rats. Tissue samples were stored at −80°C until being ready to be used for carbonylation proteomics and immunoblot assays. All surgical procedures were performed under anesthesia induced by chloral hydrate. All efforts were made to minimize suffering and distress in animals.

At least three rats from each group were used for the paraffin section sample preparation. After the above ascending aorta was infused with physiological saline, it was then perfused with 4% paraformaldehyde 0.1M phosphate buffer (pH 7.4) (4°C) 400 ml-500 ml, until the animal's liver hardened and the tail was stiff, and the perfusion was completed. Brains were taken and kept in a 4% paraformaldehyde 0.1M phosphate buffer overnight at 4°C (no more than 48 h). The next day, they were transferred to 30% sucrose and dehydrated to a low level. Then they were routinely dehydrated, waxed, paraffinized, and embedded.

### 2.4. Behavioral Monitoring

#### 2.4.1. Open-Field Experiment (OFT)

Open-field behavior apparatus: All equipment and software were purchased from SD Instruments (San Diego). The open-field device was divided into a central zone and a surrounding zone, which consists of an uncovered cuboid device of length × width × height of 100 × 100 × 40 cm, with four walls being black and the bottom being white. The bottom is divided into 25 square squares (20 × 20 cm), and each large square is subdivided into 400 small squares (1 × 1 cm). The device was placed in a room with a light intensity of 20 lux and no background noise, placing the digital camera directly above the unit. The bottom and walls of the open field were cleaned with 75% alcohol for removing odor. Each rat was preexposed for 5 min in the open-field device; then 2 tests were performed.

Open-field behavior parameters: The open-field device was divided into a central zone and the surrounding zone. Five behavioral patterns were recorded in the open-field experiments, namely, motor behavior, exploration behavior, thigmotaxic behavior, immobile-sniffing, and grooming behavior. All kinds of behaviors were mainly reflected in the central grid's staying time, number of spans, number of erections, number of cleansing, and number of defecations [19].

#### 2.4.2. Adhesion Removal Experiment

Five rats in each group were randomly selected for nasal adhesion removal and front paw adhesion removal Experiment. Referring to the method of Schallert et al. [20], training is adapted firstly and then adherent removal experiments.

### 2.5. Hematoxylin and Eosin (HE) Staining

5*μ*m slices were sectioned by 820 Rotary Tissue Slicer (AO Company, American). Then HE routine steps were as follows: dewaxing hydration, hematoxylin staining for 10–15 min, color separation with 1% hydrochloric acid alcohol, 5% eosin staining for 1 min, dehydration with gradient alcohol, clearing of tissue sections with xylene, sealing in neutral gum, and taking photos in 40× magnification with OLYMPUS BX52 Microscopic Image Acquisition System (Olympus Corporation, Japan).

### 2.6. Assessment of Apoptosis by TUNEL

The TdT-mediated end-residues, deoxyribonucleotide-terminal transferase-mediated nick end labeling (TUNEL), were used to detect the nuclear DNA fragmentation during the early stages of apoptosis, with In Situ Cell Death Detection Kit (POD) instructions. Under normal light microscope observation (OLYMPUS BX52 Microscopic Image Acquisition System and Simple PCI Microscopic Image Analysis System), the positive apoptotic cells showed brown or brown nucleus staining. Part of the cytoplasm could also be positively stained by spiking DNA fragments; normal nonapoptotic cells and negative control cell nuclei were stained. Hematoxylin is dyed blue, the nucleus is relatively large, and the shape and size are the same. The pigmented positive cells were identified as apoptotic cells according to the following criteria: a single scattered distribution; a nuclear morphology with apoptosis; no inflammatory reaction around. For positive cells lacking apoptotic nuclear morphology, astrocytes are not considered to be apoptotic cells unless their staining intensity contrasts sharply with the background and they are in a single distribution. Six sections were randomly observed per section under a light microscope (×400), and each field was at least 500 cells. The average number of positive apoptotic cells per 100 cells, i.e., the Apoptosis Index (AI), was measured using the Simple PCI microscopic image analysis software.

### 2.7. Carbonylation Proteomics

#### 2.7.1. Protein Extraction

Tissue homogenization used a Beijing Kangwei Century Technology animal cell (tissue) total protein extraction kit (No. CW0891) and an F6/10-6G ultrafine homogenizer (Fluko, Shanghai, China) in an ice bath. Hydrazide chemistry was employed to derivatize protein carbonyls from the samples (at least 3-4 samples for each concentration per treatment group) and the tissue weight was about 200 mg to extract the carbonylated protein [21]. Biotinylation was with 10% 50 mM biotin hydrazide added to a final concentration of 5 mM. The reaction was at room temperature for 2 h or at 4°C for 4 h to completely biotinylate the carbonylated proteins. To obtain protein crude extracts, samples were centrifuged at 4°C, at 3000–4000 ×* g* for 10 min; then centrifugation was repeated two to three times. Reductive amination used 5 M NaCNBH_3_ added to a final concentration of 15 mM, and the reaction was performed at 4°C for 1 h. The Schiff base was reduced to a secondary amine using a mild reducing agent, sodium cyanoborohydride, to prevent hydrolysis of the Schiff base to form a carbonyl group. The protein solution was filtered through a 0.45 *μ*m filter, and protein quantification was performed using Coomassie Brilliant Blue.

The protein solution was transferred to an ultrafiltration tube and ultrafiltered using 200 times the volume of the solution in phosphate-buffered saline (PBS) to remove the biotin hydrazide. To enrich for carbonylated proteins, 100 *μ*L of avidin beads was added to each protein sample, and the reaction was carried out at 4°C for 30 min before transferring to the Spin column. The bottom cover was removed and centrifuged at 600 ×* g* for 1 min. To separate the carbonylated proteins, after sealing the Spin column cover, 100 *μ*L PBS was added, and D-biotin was added at a final concentration of 5 mM; the reaction occurred at 4°C for 1 h. After addition to D-biotin and replacement buffer, the supernatant was transferred to a 10 kD Ultra-0.5 ultrafiltration tube and rinsed with 150–200 *μ*L of 50 mM NH_4_HCO_3_, centrifuged at 14,000 ×* g* for 10 min, and repeated three times to fully remove the D-biotin. The carbonylated proteins were collected, and after the D-biotin was sufficiently removed, 100 *μ*L of NH_4_HCO_3_ was added to the ultrafiltration tube, and the ultrafiltration tube was further rinsed with NH_4_HCO_3_ at 50 *μ*L/rinse. The carbonylated proteins were frozen and stored at 80°C. The extracted carbonylated proteins were quantified using the Coomassie Brilliant Blue assay.

#### 2.7.2. Protein Enzymolysis

Affinity-purified carbonylated proteins were concentrated in a 10 kDa Ultra-0.5 ultrafiltration tube to near dryness. For denaturation, 8 *μ*L of urea was added to 200 *μ*L of 0.1 M Tris/HCl, pH 8.5, and mixed for 1 min and then centrifuged at 14,000 ×* g* for 15 min. Disulfide bonds were disrupted using 180 *μ*L of 8 M urea and 20 *μ*L of 1 M dithiothreitol added to a final concentration of 100 mM. After shaking for 10–60 s, the incubation was performed at 56°C for 1 h. Centrifugation was at 14,000 ×* g* for 15 min. Free sulfhydryl groups were blocked by adding 100 *μ*L of freshly prepared iodoacetamide (IAA) (final concentration, 50 mM) and placing them in the dark at room temperature (or 4°C) for 30 min; then the samples were centrifuged at 14,000 ×* g* for 15 min. In addition to dithiothreitol and IAA, 100 *μ*L of 8 M urea was added, mixed for 1 min, followed by centrifugation at 14,000 ×* g* for 15 min, and the solution in the cannula was discarded twice. Displacement of urea was accomplished by adding 200 *μ*L of 50 mM ammonium bicarbonate, mixing for 1 min, and then centrifuging at 14,000 ×* g* for 30 min. The cannula solution was discarded, and rinsing was repeated two to three times [22, 23].

For enzymolysis, the separated carbonylated proteins in the Ultra-0.5 tube were ultrafiltered to dryness, then 20 *μ*L of NH_4_HCO_3_ was added to the UF tube, and the solution was transferred to a clean centrifuge tube with 10 *μ*L of NH_4_HCO_3_. The sample was rinsed twice, each time with mixing and centrifugation. The final protein solution was mixed and subsequently trypsin digested. 10× trypsin was diluted to a final concentration of 20 *μ*g trypsin in 50 *μ*L ammonium bicarbonate (100 *μ*L) and then reacted in a 37°C for 18 h. The enzymatically digested peptide samples were freeze-dried using a vacuum freeze dryer and stored at −80°C until use for mass spectrometry analyses [22, 23].

#### 2.7.3. ESI-Q-TOF-MS

The ESI-Q-TOF-MS (Brooke Inc., USA) and the liquid chromatography mass spectrometer equipped with a C_18_ reversed-phase column, capillary liquid chromatograph, and nanoliter spray source were used for mass spectrometry. The ESI-Q-TOF-MS spectra were measured on a micrOTOF-Q II mass spectrometer (Bruker Daltonics, Fremont, CA, USA) using nanocolumns (75 mm × 150 mm; Dionex, Sunnyvale, CA, USA) in conjunction with nano-liquid chromatography (LC) (Ultimate 3000; Dionex) for protein identification (nanoLC-MS/MS) with an autosampler pump.

The digested peptide mixture produced by trypsin hydrolysis was injected into the mass spectrometer from the autosampler dish. The ESI source was as follows: 1.2 kV, desolvation temperature, 150°C. The sample was introduced with a flow rate of 0.3 *μ*L/min using a water/acetonitrile gradient. All mass spectra were in positive ion mode and the collision gas was argon for MS/MS measurements. The instrument calibration range was 100–3000 m/Z and the external calibration standard (Tunemix; Bruker) was supplied by Agilent Technologies, Santa Clara, CA, USA. MS and MS/MS data were automatically collected and processed by Bruker 4.0 software (Bruker).

#### 2.7.4. Carbonylation Proteomics Data Analyses and Bioinformatics

The MS data were searched by using the database of the MASCOT server (version 2013, Matrix Science-Mascot-MS/MS-Ions Search). The parameters included the Swiss-Prot/Uniprot database, and trypsin lysis, which allowed one uncut site. The species were as follows: rat; MS/MS fragment ion mass error set to 0.2 Da. Because Mascot has a limit of nine modifications per search, the variable modifiers were searched multiple times and each sample contained a maximum of six modifiers at a time. According to Madian et al. [23], the oxidative modification sites of carbonylated proteins were searched in several steps. The first modification was fixed as follows: cysteine was iodoacetamidated to carbamidomethyl (C) without variable modification; the second step was fixed. Modifications included carbamidomethyl (C), variably modified to oxidation or hydroxylation (C-terminal G, D, F, HW, K, M, N, R, Y). Then, changes in the variable modification from cysteine sulfenic acid-tryptophan oxidation to oxolactone were performed. The term “keratin filaments” appears in the Gene Ontology (GO) Cellular Component Ontology, and “Keratin, type I cytoskeletal 15” was included in the gene name. Protein abundance was based on the protein-rich fraction database (PaxDb2.0), and protein function and subcellular localization were searched through the Uniprot database. Information on signal pathway analysis of carbonylated proteins was obtained using the GeneMANIA Prediction Server (Version 2.8). The carbonyl protein network was obtained from the GeneMANIA Cytoscape plugin (version 3.2) and STRING9.0 multiple name analysis software.

### 2.8. Western Blot Analysis of CaMKII *α* and *β*

We used the BCA Protein Assay Kit (Wellbio, American Diagnostica Inc., Bloomberg, USA) to determine total protein concentration and performed the assay according to the manufacturer's instructions. Total cellular protein extracts were separated on 10% SDS-polyacrylamide gel and transferred to PVDF membranes. A T-Pro prestained protein ladder was used as a molecular marker to estimate the size of the proteins. The membranes were incubated with the primary antibodies, anti-CaMK II *β*(~55 kD,11533-1-AP, Proteintech Group, USA), anti-CaMK II**α** (~55 kD, NO.20666-1-AP, Proteintech Group, USA), and anti-GAPDH (~36kDa, NO.10494-1-AP, Proteintech Group, USA) followed by an HRP-conjugated secondary antibody (Proteintech). The separated proteins were detected by developing the PVDF membranes (NO. 10285-1-AP, Proteintech) with a ChemiLucent ECL Detection System (Millipore, Billerica, MA, USA). The exposed X-ray films were scanned using the Tanon Gel Image Shooting System (Tanon, Shanghai, China), and data analysis was performed on the Tanon Gel Image Processing System and Image J (National Institute of Mental Health, Bethesda, Maryland, USA).

### 2.9. Real-Time Quantitative Polymerase Chain Reaction (RT-qPCR) Detection of Target mRNA

Total RNA was extracted using the TRIzol (Invitrogen, Carlsbad, California, USA) and miRNeasy mini kit (QIAGEN, Hilden, Germany) kits, which effectively covers all types of RNA (Table 1). RNA solution was determined by UV spectrophotometer (ND-1000, Nanodrop Technologies, Wilmington, USA) and the ratio of 260/280 (the purity of RNA) ranged from 1.8 to 2.1, indicating that the RNA solution can be used in the next experiment.

Analyzed from the amplification profile and dissolution profile of the target mRNA by RT-qPCR, it is shown that the amplification efficiency was high, the Tm value was of uniform nature and specificity, and there were no nonspecific amplification and dimer formation.

At least three samples were randomly assigned in each group, and the RT-qPCR was performed for confirming the amplification curve and melting curve, and recording the Ct (cycle threshold) value of each gene mRNA. The mRNA expression of the target gene was quantitatively determined by using the method of 2-delta Ct as the internal reference.

(1) Target  gene  mRNA  relative  quantity=2−∆∆Ct∆∆Ct=Cttarget−Ctβ−actinSamples−Cttarget−Ctβ−actinControl

### 2.10. Statistical Analysis

Data from all experiments are presented as mean ± SEM. Statistical analysis was performed using predictive analytics software statistics 16.0 (SPSS Inc., Chicago, IL, USA). Comparisons across the experimental groups were performed using one-way analysis of variance (ANOVA). The results of behavior experiments were analyzed by repeated measures of variance. Statistical significance of the effects of the experimental treatment was determined by comparing the areas under the curve (p<0.05).

## 3. Results

### 3.1. Behavioral Monitoring Indicators in Aging Rats

#### 3.1.1. Results of Open-Field Experiments

The open-field experiment is a sensitive method commonly used in animal experiments to detect dopaminergic function activity deficiency [24]. Central lattice retention time was significantly lower in the M-EX as compared to the M-SED group (P < 0.01). On the other hand, the number of spans, erections, and cleansing episodes was higher in the M-EX than in the M-SED group (P < 0.05), while there was no significant difference in defecation (P > 0.05) (Table 2).

#### 3.1.2. Results of Adhesion Removal Experiment

The detection of adhesion removal experiment when detecting motor defects may be a more sensitive experimental method than the field test and is often used to detect mild striatal pathway dysfunction [20]. The nasal adhesion removal experiment was completed within 3 min (Table 3). The time to remove the nasal adhesions was shorter in the M-EX than in the M-SED group (P < 0.01). All rats removed the adhesive material from their forepaws within 5 min, but the time taken was longer in the M-EX than in the M-SED group (P < 0.01).

### 3.2. HE Staining and Microscopic Imaging of the Striatum

**Figure 1:**
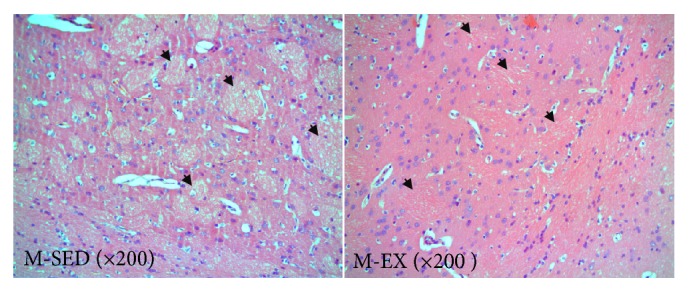
Microscopic images of HE staining. M-SED, middle-aged sedentary control group; M-EX, middle-aged aerobic exercise runner group; HE, haematoxylin and eosin (arrows indicate the striatum matrix.).

A histological analysis of HE-stained striatal tissue sections revealed that the matrix was clearly separated and the gap between the matrix and striatum was smaller in the M-SED than in the M-EX group, in which the matrix was more compact and the gap was larger (Figure 1).

### 3.3. Results of the TUNEL Assay

**Figure 2:**
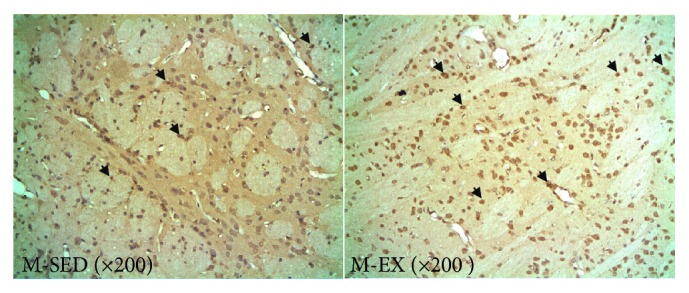
Microscopic images of TUNEL assay (arrows indicate apoptotic nuclei). M-SED, middle-aged sedentary control group; M-EX, middle-aged aerobic exercise runner group; TUNEL, TdT-mediated dUTP nick end labeling.

Normal nuclei in the striatum were stained blue whereas apoptotic nuclei appeared brown. There were more apoptotic nuclei in the M-SED than in the M-EX group (Figure 2). Some apoptotic nuclei appeared vacuolated and some in the M-EX group appeared follicular with a darker color.

Apoptotic nuclei were detected in the rat striatum; the rate of apoptosis was 100% (Table 4). The apoptotic index was 57% higher in the M-EX than in the M-SED group (P < 0.01).

### 3.4. Differential Carbonyl Proteomics Analysis

The ESI-Q-TOF-MS/MS analysis identified 36 carbonylated proteins with oxidation modification sites in both the M-SED and M-EX groups. There were 17 and 19 carbonylated proteins with such sites that were specific to the M-SED and M-EX groups, respectively, whereas 40 of the proteins were common to both groups (Table 5).

Carbonylated proteins with oxidation modification sites that were unique to the M-SED group included CaMKII*β*, synaptosomal-associated protein 25 (Snap25), synapsin-2 (Syn2), heterogeneous nuclear ribonucleoprotein (Hnrnp)a2b1, complement component 1q subunit-like protein (C1QBP), G protein/guanylate binding protein (GnB)4, T-complex protein 1 subunit epsilon (Cct5), and neuromodulin (Gap43). Those specific to the M-EX group included isocitrate dehydrogenase [NAD] subunit alpha (Idh3a), synaptophysin (Syp), ubiquitin carboxyl-terminal hydrolase isozyme (UCH)-L1, elongation factor 1 (Eef1a1), acid aldolase A fructose-diphosphate aldolase (ALDOA), myelin-oligodendrocyte glycoprotein (MOG), and malate dehydrogenase (MDH)2 (Table 5).

CAMKII*β* is a component of the N-methyl-d-aspartate receptor (NMDAR) complex in excitatory synapses and may regulate NMDAR-dependent potentiation of the *α*-amino-3-hydroxy-5-methyl-4-isoxazolepropionic acid receptor and synaptic plasticity. HnRNPs bind to neonatal transcripts and are involved in messenger RNA biosynthesis, DNA repair, telomere biogenesis, cell signaling, and gene expression regulation. Studies have shown that Hnrnpa2b1 is closely related to the Ras-Raf-mitogen-activated protein kinase (MAPK)-ERK signaling pathway [25].

**Figure 3:**
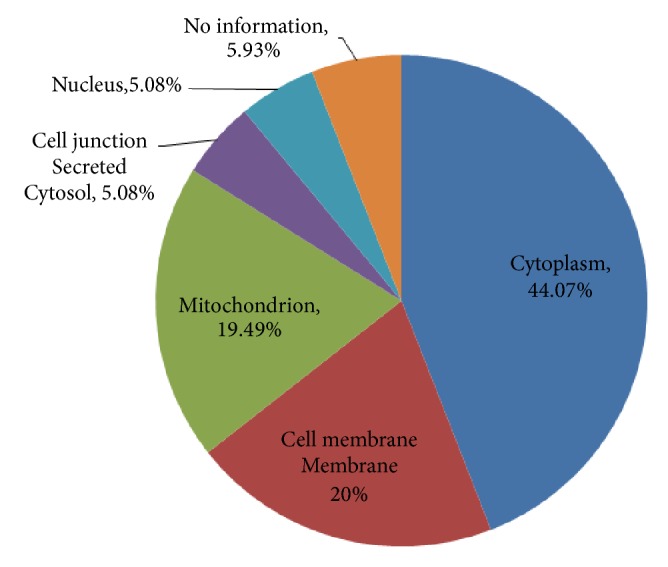
Subcellular localization of carbonylated proteins in rats' striatum.

A search of the Swiss-Prot/Uniprot database revealed the localization of the identified carbonylated proteins as cytoplasm (44.07%), mitochondria (19.49%), cell membrane (20.34%), nucleus (5.08%), junction/secreted/cytosol (5.08%), and no information (5.93%) (Figure 3).

Protein carbonyl modifications include (1) carbonyl groups that can be oxidized by amino acid side chains (proline, arginine, lysine, threonine, glutamic acid, and aspartate residues) that are directly formed in proteins or the *α*-amide or diamide pathway oxidatively cleaving the polypeptide backbone; (2) proteins that can also be an indirect carbonylation product of lipid peroxidation, such as 4-hydroxy-2-nonenal, 2-propionaldehyde, and malondialdehyde (Michael and/or Schiff bases are added to cysteine, histidine, or lysine residues); and (3) the formation of advanced glycation end products and free radical-oxidized proteins that formed carbonylated proteins. These proteins can be oxidized in more than 35 ways and all three of these basic posttranslational modifications can be distinguished by mass spectrometry. We found that oxidatively modified sites involved 28 species, as follows: Oxidation (C-term G), Oxidation (D), Oxidation (F), Oxidation (HW), Oxidation (K), Oxidation (M), Oxidation (N), Oxidation (R), Oxidation (Y) [oxidation or hydroxylation]; Oxidation (C) [cysteine sulfenic acid]; Oxidation (P) [proline oxidation to glutamic semialdehyde]; Dioxidation (C) [sulfinic acid]; Dioxidation (F) [phenylalanine oxidation to dihydroxyphenylalanine]; Dioxidation (M) [sulphone]; Dioxidation (W) [tryptophan oxidation to formylkynurenine]; Dioxidation (K), Dioxidation (P), Dioxidation (R) [dioxidation or dihydroxy]; Trioxidation (C) [cysteine oxidation to cysteic acid]; HNE (C), HNE (H), HNE (K) [4-hydroxynonenal Michael adduct]; glucosone (R) [condensation product of glucosone]; Lys->Allysine (K) [lysine oxidation to aminoadipic semialdehyde]; 3-deoxyglucosone (R) [3-deoxyglucosone adduct]; Arg->GluSA (R) [arginine oxidation to glutamic semialdehyde]; Delta:H(2)C(5) (K) [adduct formed from malondialdehyde (MDA) ]; 4-ONE (C) [4-Oxononenal]; Arg biotin hydrazide (R) [oxidized arginine biotinylated with biotin hydrazide]; Lysbiotinhydrazide (K) [oxidized lysine biotinylated with biotin hydrazide]; OxLysBiotin (K) [oxidized lysine biotinylated with biotin-LC-hydrazide]; probiotinhydrazide (P) [oxidized proline biotinylated with biotin hydrazide]; Thrbiotinhydrazide (T) [oxidized Threonine biotinylated with biotin hydrazide]; Didehydro (T) [2-amino-3-oxo-butanoic_acid]; Pro->pyro-Glu (P) [proline oxidation to pyroglutamic acid]; Pro->Pyrrolidinone (P) [proline oxidation to pyrrolidinone]; Trp->Hydroxykynurenine (W) [tryptophan oxidation to hydroxykynurenine]; Trp->Kynurenin (W) [tryptophan oxidation to kynurenine]; Trp->Oxolactone (W) [tryptophan oxidation to oxolactone].

### 3.5. Screening Target Genes from Differentially Carbonylated Proteins

**Figure 4:**
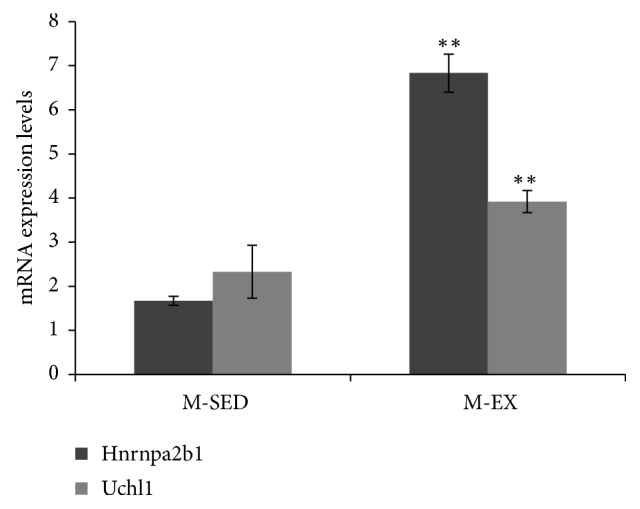
mRNA expression levels of Hnrnpa2b1 and UCH-L1 as determined by RT-qPCR. M-SED, middle-aged sedentary control group; M-EX, middle-aged aerobic exercise runner group; ^*∗*  *∗*^*ρ* < 0.05 ^*∗*  *∗*^*ρ* < 0.01 vs. M-SED; Hnrnpa2b1, heterogeneous nuclear ribonucleoproteins A2/B1; UCH-L1, ubiquitin carboxyl-terminal hydrolase isozyme L1.

The results revealed that further studies are needed to elucidate the role of protein carbonylation of Hnrnpa2b1 and UCH-L1. The mRNA expression levels of UCH-L1 and Hnrnpa2b1 were significantly upregulated in the M-EX group, as compared with the M-SED group (P < 0.01; Figure 4).

### 3.6. Screening of the CaMK Pathway by Differential Carbonylation Proteomics

The carbonylated proteins with oxidation modification sites unique to the M-SED group included CaMKII*β*. CaMKII*β* and CaMKII*α* are of the most abundant protein kinases in the brain that regulate calcium signaling, a critical molecule in multiple signal transduction pathways, and play a key role in synaptic plasticity, learning, and memory [26]. In addition, they are highly expressed in a specific subcellular localization in neurons, wherein they convert the intracellularly elevated calcium signals to a range of target proteins, including ion channels and transcriptional activators; the synaptic transmission has regulatory roles in downstream signaling pathway-associated proteins [27].

**Figure 5:**
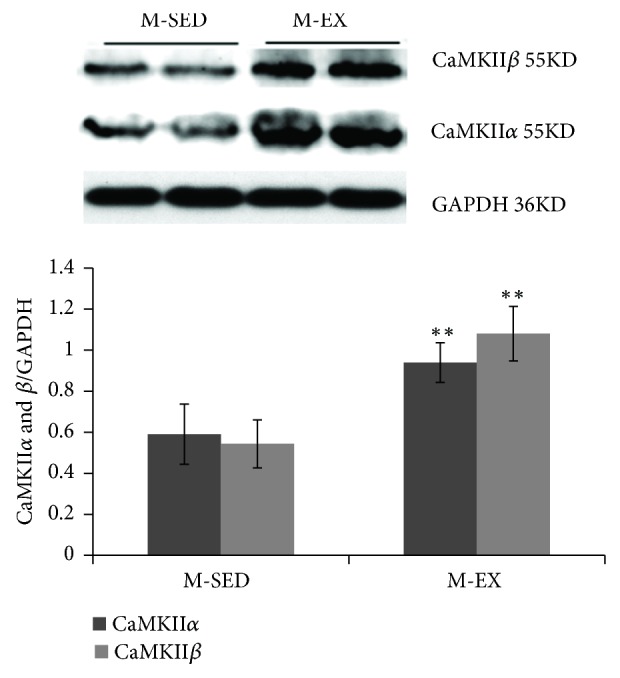
CaMKII*α* and *β* expression in the rat striatum. M-SED, middle-aged sedentary control group; M-EX, middle-aged aerobic exercise runner group; ^*∗*  ^*ρ* < 0.05 ^*∗*  *∗*^*ρ* < 0.01 vs. M-SED; CaMKII *α* and *β*, Ca^2+^/calmodulin-dependent protein kinases *α* and *β*; GAPDH, glyceraldehyde-3-phosphate dehydrogenase.

**Figure 6:**
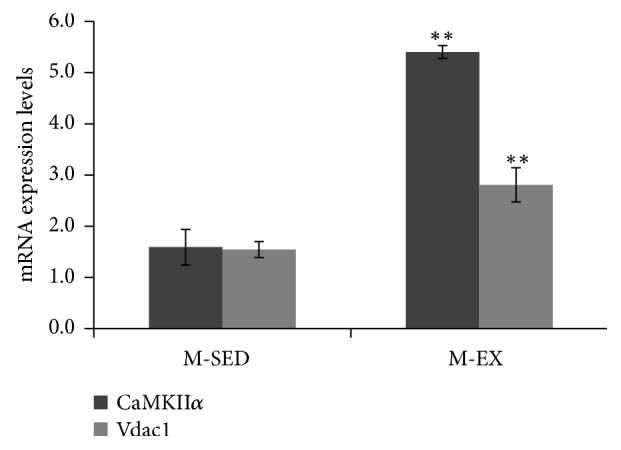
mRNA expression levels of CaMKII*α* and Vdac1 by RT-qPCR. M-SED, middle-aged sedentary control group; M-EX, middle-aged aerobic exercise runner group; ^*∗*^*ρ* < 0.05 ^*∗*  *∗*^*ρ* < 0.01 vs. M-SED; CaMKII*α*, Ca^2+^/calmodulin-dependent protein kinases or CaM kinases *α*; Vdac1, voltage-dependent anion-selective channel protein 1.

CaMKII *α* and *β* protein level was upregulated in the M-EX as compared to the M-SED group (P < 0.01; Figure 5). Additionally, CaMKII*α* and voltage-dependent anion-selective channel protein (VDAC)1 mRNA levels were upregulated in the M-EX as compared to the M-SED group (P < 0.01; Figure 6).

### 3.7. Screening of the Phosphoinositide 3-Kinase/Protein Kinase B/Mammalian Target of Rapamycin (PI3K/Akt/mTOR) Pathway by Differential Carbonylation Proteomics

mTOR not only regulates cell proliferation and survival but also participates in membrane transport and protein degradation, especially in the regulation of protein translation levels. Recently, rapamycin has been shown to promote autophagy and apoptosis, remove abnormal proteins such as amyloid polypeptide in AD and mutated *α*-synuclein in familial PD, and thus treat related diseases [28].

**Figure 7:**
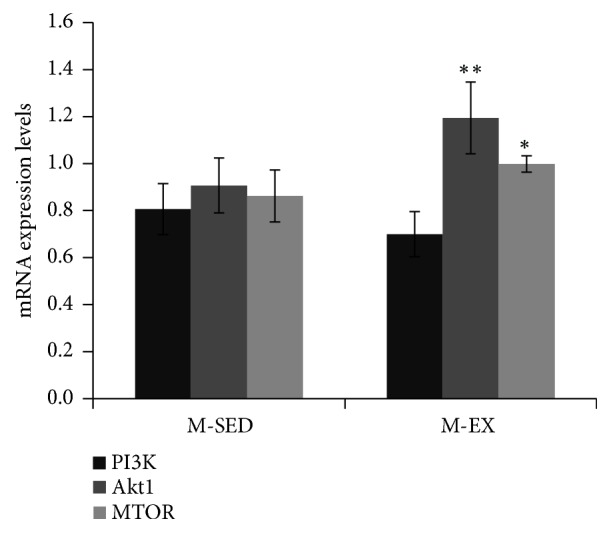
mRNA expression levels of PI3K, Akt, and mTOR, as determined by RT-qPCR. M-SED, middle-aged sedentary control group; M-EX, middle-aged aerobic exercise runner group; ^*∗*  ^*ρ* < 0.05 ^*∗*  *∗*^*ρ* < 0.01 vs. M-SED; PI3K, phosphoinositide 3-kinase; Akt, protein kinase B; and mTOR, mammalian target of rapamycin.

PI3K mRNA level was downregulated (P > 0.05) whereas the mRNA levels of Akt (P < 0.01) and mTOR (P < 0.05) were upregulated in the M-EX as compared to the M-SED group (Figure 7).

## 4. Discussion

### 4.1. Effects of Regular Aerobic Exercise on Protein Carbonylation in the Rat Striatum

The ESI-Q-TOF-MS/MS analysis identified 36 carbonylated proteins in the rat striatum, of which 17 and 19 were specific to the M-SED and M-EX groups, respectively. The carbonylated proteins were related to energy metabolism, mitochondrial inner membrane, ATPase activity, development, synaptic conduction, axonal targeting, and aging, suggesting that protein carbonylation is selective and that the relative amount of protein does not determine the degree of modification. Protein carbonylation increases with aging and is especially abundant in elderly individuals; nearly one-third of all proteins are carbonylated [29, 30]. Erroneously translated proteins are prone to and become less stable after carbonylation [31].

Carbonylated proteins specific to the M-SED group included CaMKII*β*, Snap25, Syn2, Hnrnpa2b1, and Gap43. On the other hand, CaMKII*β* and Hnrnpa2b1 mRNA levels were upregulated in the M-EX group. HNE inhibits oxidant-induced Ca^2+^ release from the mitochondria in a concentration-dependent manner (10–50 *μ*M) [32, 33]; this requires oxidation of NADH to NAD^+^, which in turn hydrolyzes NAD^+^ to niacinamide and mono-ADP-ribose following transient binding of mono ADP-ribose to mitochondrial proteins. Inhibition of pyridine nucleotide hydrolysis by HNE results in Ca^2+^ overload in mitochondria and inhibition of Ca^2+^-dependent mitochondrial enzymes, leading to disruption of the cell cycle, and inhibition of DNA synthesis and cell proliferation [34, 35].

Carbonylated proteins unique to the M-EX group included Idh3a, Syp, Uchl1, Eef1a1, ALDOA, MOG, and MDH2. Interestingly, the mRNA levels of UCH-L1 were upregulated in this group, suggesting that regular aerobic exercise modulates proteins associated with mitochondrial and striatum development-related signaling transduction proteins. However, additional studies are needed to clarify the specific mechanisms involved. Exercise leads to stress (oxidative and ischemia-reperfusion) mainly from differences in energy metabolism and demand [36, 37].

### 4.2. Effects of Regular Aerobic Exercise on Apoptosis and Regulation of the CaMK/mTOR Signaling Pathway in the Rat Striatum

Accumulation of carbonylated proteins can lead to polymerization of damaged proteins, dysregulation of cellular function, and other pathophysiological changes that accelerate aging. Proteins that are overoxidized and cross-linked cannot be degraded by the proteasome, possibly due to structural changes that render the catalytic site of the enzyme complex unrecognizable or inaccessible, eventually leading to apoptosis [38]. In the present study, we detected apoptotic nuclei in the rat striatum; the rate of apoptosis was 100%, and the apoptotic index of the striatum was 57% higher in the M-EX as compared to the M-SED group.

CaMKII, among the most abundant protein kinases in the brain, regulates calcium signaling and plays an important role in multiple signal transduction pathways involved in synaptic plasticity, learning, and memory [26, 39]. We found here that carbonylation of CaMKII*β* was specific to the M-SED group, whereas the CaMK pathway-related genes CaMKII*α* and VDAC1 were significantly increased in the M-EX group, indicating that regular aerobic exercise is beneficial for maintaining calcium regulation. CaMKII is an important component of postsynaptic densities, accounting for 30%–50% of all synaptic proteins [40]; it is thought to play an important role in long-term memory. CaMKII phosphorylates synaptophysin I, which then fuses to the presynaptic membrane and stimulates neurotransmitter release [41]. Maintaining intracellular calcium homeostasis is critical for neuronal functions; a perturbation of this process can lead to cell damage or death [42, 43].

The PI3K/Akt/mTOR signaling pathway is involved in cell survival [44], as well as cell proliferation, apoptosis, and differentiation and metabolism [45, 46]. Interestingly, Akt and mTOR transcript levels were upregulated by regular aerobic exercise, which is known to induce CaMK and PI3K/Akt/mTOR signaling pathways to prevent excessive apoptosis in the rat striatum as a neuroprotective mechanism. In the injured spinal cord, treadmill exercise promoted the recovery of motor function by suppressing apoptosis [47]. The beneficial effects of exercise may be attributed to the increased release of neurotrophic factors via activation of the PI3K/Akt pathway: exercise after brain injury increased the production of nerve growth factor and brain-derived neurotrophic factor and the activation of TrkA/B receptor, which activated the PI3K/Akt pathway and inhibited apoptosis of hippocampal neurons [48]. Long-term regular aerobic exercise itself is a stress stimulus [17]; however, a moderate amount of physical activity strengthens the body [49–51] and can improve the functioning of various organs, reduce the incidence of disease, and improve quality of life [52, 53].

## 5. Conclusions

Regular aerobic exercise for 10 weeks (incremental for the first 6 weeks followed by constant loading for 4 weeks) enhanced carbonylation of CaMKII*β* and Hnrnpa2b1 and modulated apoptosis via activation of CaMK and phosphoinositide 3-kinase/protein kinase B/mTOR signaling. It also promoted normal apoptosis in the rat striatum, which may have protective effects in neurons.

## Figures and Tables

**Table 1:** Primers for RT-qPCR.

Target gene	Accession number	Sequence (5′—3′)
*β*-actin		F: GAGATTACTGCTCTGGCTCCTA
		R: GGACTCATCGTACTCCTGCTTG
CaMK IIa	KCC2A_RAT	GeneCopoeia, Inc, USA Catalog#: RQP049239
Vdac1	VDAC1_RAT	GeneCopoeia, Inc, USA Catalog#: RQP051105
Itpr1	PI3R_RAT	GeneCopoeia, Inc, USA Catalog#: RQP045271
Akt1	Akt1_RAT	GeneCopoeia, Inc, USA Catalog#: RQP051482
mTOR	mTOR_RAT	GeneCopoeia, Inc, USA Catalog#: RQP050125
UCH-L1	UCH-L1_RAT	GeneCopoeia, Inc, USA Catalog#: RQP049756
Hnrnpa2b1	ROA2_RAT	GeneCopoeia, Inc, USA Catalog#: RQP055531

**Table 2:** The results of open-field experiments.

Groups	Central grid's staying time (s)	Spans	Erections	Cleansing	Defecations
M-SED	17.60±8.36	17.40±9.95	4.20±1.93	1.80±0.37	1.00±0.63
M-EX	6.40±3.34^*∗∗*^	23.60±9.64^*∗*^	7.00±2.81^*∗*^	7.60±1.96^*∗*^	1.00±0.45

M-SED, middle-aged sedentary control group; M-EX, middle-aged aerobic exercise runner group; *∗ρ* < 0.05 *∗∗ρ* < 0.01 vs. M-SED.

**Table 3:** The results of adhesion removal experiment.

Groups	The nasal adhesion removal experiment (s)	The paw adhesion removal experiment (s)
M-SED	26.13±7.42	8.97±1.86
M-EX	10.56±2.47^*∗∗*^	15.41±2.82^*∗∗*^

M-SED, middle-aged sedentary control group; M-EX, middle-aged aerobic exercise runner group; ^*∗*^*ρ* < 0.05 ^*∗∗*^*ρ* < 0.01 vs. M-SED.

**Table 4:** Apoptosis rates as determined by the TUNEL assay.

Groups	apoptosis index (AI)
M-SED	7.00±2.71
M-EX	10.99±2.93^*∗∗*^

M-SED, middle-aged sedentary control group; M-EX, middle-aged aerobic exercise runner group; ^*∗*^*ρ* < 0.05 ^*∗∗*^*ρ* < 0.01 vs. M-SED; AI, apoptotic index.

**Table 5:** The screening of the different carbonylated proteins in rats' striatum.

Accession number	carbonylated proteins	M-SED				M-EX			
		Score	Matches	Sequences	Unique	Score	Matches	Sequences	Unique
AT12A_RAT^a^	Potassium-transporting ATPase alpha chain 2, GN=Atp12a	*52*	*4(1)*	*3(1)*	*2*	46	4(1)	3(1)	1
AT1B1_RAT^a^	Sodium/potassium-transporting ATPase beta-1, GN=Atp1b1	*122*	*9(3)*	*5(2)*	*9*	113	5(2)	4(2)	5
GBB2_RAT^a^	Guanine nucleotide-binding protein G(I)/G(S)/G(T) subunit beta-2, GN=Gnb2	*120*	*4(3)*	*3(2)*	*3*	63	5(2)	2(2)	5
GNAO_RAT^a^	Guanine nucleotide-binding protein G(o) subunit alpha OS=Rattus norvegicus GN=Gnao1 PE=1 SV=2	*317*	*14(10)*	*7(5)*	*14*	70	3(2)	3(2)	3
KCC2B_RAT^a^	Calcium/calmodulin-dependent protein kinase type II subunit beta, GN=CaMKIIb	*69*	*9(4)*	*5(4)*	*5*	168	10(7)	6(4)	4
TBB3_RAT^a^	Tubulin beta-3 chain, GN=Tubb3	*2062*	*66(52)*	*20(17)*	*17*	919	26(20)	14(13)	5
AT1A2_RAT^a^	Sodium/potassium-transporting ATPase subunit alpha-2, GN=Atp1a2	*488*	*16(12)*	*8(7)*	*8*	ND	ND	ND	ND
C1QBP_RAT^a^	Complement component 1 Q subcomponent-binding protein, mitochondrial OS=Rattus norvegicus GN=C1qbp PE=1 SV=2	*58*	*4(3)*	*3(2)*	*4*	ND	ND	ND	ND
COX5A_RAT^a^	Cytochrome c oxidase subunit 5A, GN=Cox5a	*25*	*4(1)*	*2(1)*	*4*	ND	ND	ND	ND
COX5B_RAT^a^	Cytochrome c oxidase subunit 5B, GN=Cox5b	*60*	*6(3)*	*3(2)*	*6*	ND	ND	ND	ND
GBB1_RAT^a^	Guanine nucleotide-binding protein G(I)/G(S)/G(T) subunit beta-1,GN=Gnb1	*87*	*4(3)*	*4(3)*	*3*	ND	ND	ND	ND
GBB4_RAT^a^	Guanine nucleotide-binding protein subunit beta-4, GN=Gnb4	*76*	*3(2)*	*2(1)*	*1*	ND	ND	ND	ND
NEUM_RAT^a^	Neuromodulin, GN=Gap43	*20*	*7(1)*	*2(1)*	*7*	ND	ND	ND	ND
ROA2_RAT^a^	Heterogeneous nuclear ribonucleoproteins A2/B1,GN=Hnrnpa2b1	*56*	*3(1)*	*3(1)*	*3*	ND	ND	ND	ND
SNP25_RAT^a^	Synaptosomal-associated protein 25,GN=Snap25	*64*	*6(1)*	*6(1)*	*6*	ND	ND	ND	ND
SYN2_RAT^a^	Synapsin-2,GN=Syn2	*57*	*4(1)*	*4(1)*	*4*	ND	ND	ND	ND
TCPE_RAT^a^	T-complex protein 1 subunit epsilon,GN=Cct5	*46*	*2(1)*	*2(1)*	*2*	ND	ND	ND	ND
1433E_RAT^b^	14-3-3 protein epsilon, GN=Ywhae	66	5(3)	2(2)	5	*56*	*5(4)*	*3(3)*	*5*
1433B_RAT^b^	14-3-3 protein beta/alpha, GN=Ywhab	ND	ND	ND	ND	*43*	*5(2)*	*4(2)*	*4*
ACTN1_RAT^b^	Alpha-actinin-1,GN=Actn1	ND	ND	ND	ND	*57*	*8(2)*	*8(2)*	*8*
ACTC_RAT^b^	Actin, alpha cardiac muscle 1,GN=Actc1	682	30(20)	11(6)	9	*470*	*22(14)*	*11(7)*	*10*
ALDOA_RAT^b^	Fructose-bisphosphate aldolase A,GN=Aldoa	ND	ND	ND	ND	*46*	*5(2)*	*4(2)*	*5*
ARF1_RAT^b^	ADP-ribosylation factor 1,GN=Arf1	ND	ND	ND	ND	*45*	*3(1)*	*2(1)*	*3*
DREB_RAT^b^	Drebrin, GN=Dbn1	ND	ND	ND	ND	*36*	*2(1)*	*2(1)*	*2*
EF1A1_RAT^b^	Elongation factor 1-alpha 1,GN=Eef1a1	ND	ND	ND	ND	*48*	*3(1)*	*3(1)*	*3*
HXK1_RAT^b^	Hexokinase-1,GN=Hk1	ND	ND	ND	ND	*46*	*6(2)*	*5(1)*	*6*
IDH3A_RAT^b^	Isocitrate dehydrogenase [NAD] subunit alpha, GN=Idh3a	ND	ND	ND	ND	*29*	*4(1)*	*4(1)*	*4*
KAD1_RAT^b^	Adenylate kinase isoenzyme 1,GN=Ak1	ND	ND	ND	ND	*45*	*2(1)*	*2(1)*	*2*
MDHC_RAT^b^	Malate dehydrogenase, GN=Mdh1	ND	ND	ND	ND	*28*	*4(1)*	*4(1)*	*4*
MDHM_RAT^b^	Malate dehydrogenase, GN=Mdh2	ND	ND	ND	ND	*78*	*3(1)*	*3(1)*	*3*
MOG_RAT^b^	Myelin-oligodendrocyte glycoprotein, GN=Mog	ND	ND	ND	ND	*54*	*3(1)*	*3(1)*	*3*
MYH10_RAT^b^	Myosin-10,GN=Myh10	ND	ND	ND	ND	*27*	*12(1)*	*12(1)*	*12*
PROF2_RAT^b^	Profilin-2,GN=Pfn2	ND	ND	ND	ND	*17*	*2(0)*	*2(0)*	*2*
SUCA_RAT^b^	Succinyl-CoA ligase [ADP/GDP-forming] subunit alpha, GN=Suclg1	ND	ND	ND	ND	*24*	*2(0)*	*2(0)*	*2*
SYPH_RAT^b^	Synaptophysin, GN=Syp	ND	ND	ND	ND	*24*	*4(0)*	*3(0)*	*4*
UCHL1_RAT^b^	Ubiquitin carboxyl-terminal hydrolase isozyme L1,GN=Uchl1	ND	ND	ND	ND	*41*	*3(1)*	*3(1)*	*3*

M-SED, middle-aged sedentary control group; M-EX, middle-aged aerobic exercise runner group; letter a is M-SED; letter b is M-EX.

## Data Availability

The data used to support the findings of this study are available from the corresponding author upon request.
